# Activation of the omega-3 fatty acid receptor GPR120 mediates anti-inflammatory actions in immortalized hypothalamic neurons

**DOI:** 10.1186/1742-2094-11-60

**Published:** 2014-03-27

**Authors:** Leigh Wellhauser, Denise D Belsham

**Affiliations:** 1 Department of Physiology, Faculty of Medicine, University of Toronto, 1 King’s College Circle, Medical Sciences Building 3344, Toronto, Ontario M5S 1A8, Canada; 2 Departments of Obstetrics, Gynaecology and Medicine, University of Toronto, 92 College Street, Toronto, Ontario M5G 1 L4, Canada; 3 Division of Cellular and Molecular Biology, Toronto General Hospital Research Institute, University Health Network, 610 University Avenue, Toronto, Ontario M5G 2 M9, Canada

**Keywords:** Inflammation, Hypothalamus, Signal transduction, Neuronal cell line, Tumor necrosis factor α, Docosahexaenoic acid

## Abstract

**Background:**

Overnutrition and the ensuing hypothalamic inflammation is a major perpetuating factor in the development of metabolic diseases, such as obesity and diabetes. Inflamed neurons of the CNS fail to properly regulate energy homeostasis leading to pathogenic changes in glucose handling, feeding, and body weight. Hypothalamic neurons are particularly sensitive to pro-inflammatory signals derived locally and peripherally, and it is these neurons that become inflamed first upon high fat feeding. Given the prevalence of metabolic disease, efforts are underway to identify therapeutic targets for this inflammatory state. At least in the periphery, omega-3 fatty acids and their receptor, G-protein coupled receptor 120 (GPR120), have emerged as putative targets. The role for GPR120 in the hypothalamus or CNS in general is poorly understood.

**Methods:**

Here we introduce a novel, immortalized cell model derived from the rat hypothalamus, rHypoE-7, to study GPR120 activation at the level of the individual neuron. Gene expression levels of pro-inflammatory cytokines were studied by quantitative reverse transcriptase-PCR (qRT-PCR) upon exposure to tumor necrosis factor α (TNFα) treatment in the presence or absence of the polyunsaturated omega-3 fatty acid docosahexaenoic acid (DHA). Signal transduction pathway involvement was also studied using phospho-specific antibodies to key proteins by western blot analysis.

**Results:**

Importantly, rHypoE-7 cells exhibit a transcriptional and translational inflammatory response upon exposure to TNFα and express abundant levels of GPR120, which is functionally responsive to DHA. DHA pretreatment prevents the inflammatory state and this effect was inhibited by the reduction of endogenous GPR120 levels. GPR120 activates both AKT (protein kinase b) and ERK (extracellular signal-regulated kinase); however, the anti-inflammatory action of this omega-3 fatty acid (FA) receptor is AKT- and ERK-independent and likely involves the GPR120-transforming growth factor-β-activated kinase 1 binding protein (TAB1) interaction as identified in the periphery.

**Conclusions:**

Taken together, GPR120 is functionally active in the hypothalamic neuronal line, rHypoE-7, wherein it mediates the anti-inflammatory actions of DHA to reduce the inflammatory response to TNFα.

## Background

Hypothalamic inflammation in reaction to excessive nutrients and the consequent innate immune response is a leading contributor to diet-induced obesity (DIO) and type 2 diabetes mellitus (T2DM). Although systemic levels of proinflammatory cytokines are elevated in these conditions, it remains unclear whether peripheral cytokines that cross the blood-brain barrier (BBB) contribute significantly to the inflammatory state of the central nervous system (CNS) [1-3]. Regardless, the CNS maintains a functional innate immune system and expresses equivalent cytokines and receptors, supplying the CNS with a plethora of locally derived proinflammatory signals [4-7]. In fact these cytokines, particularly from the hypothalamus, are the first to be identified in the early stages of metabolic diseases, implicating these proinflammatory signals as predictors or perpetuators of impending pathology [8-10].

Inflammation in the CNS disrupts energy homeostasis by impairing insulin sensitivity, glucose sensing, and fatty acid utilization, as well as disrupting the expression of neuropeptides linked to feeding [4,6,10-12]. In part, energy deregulation occurs through activation of the canonical inflammatory pathway, inhibitor of the IkappaB kinase beta/nuclear factor kappa B (IKK-β/NF-κB) cascade, which is highly conserved, fully functional, and whose components are greatly enriched in the mediobasal hypothalamus [6,13-15]. Genetic ablation of IKK-β/NF-κB signaling in neurons by impairing IKK-β activity is sufficient to reduce food intake, body weight gain, and glucose intolerance typically observed in high fat diets, thus highlighting this pathway as a promising therapeutic target [10,14,16].

Beyond genetic ablation, abrogation of endogenous IKK-β/NF-κB signaling is also achieved by the activation of a G-protein coupled receptor (GPR) of the rhodopsin family and also a bona fide long-chain fatty acid (FA) sensor coined GPR120 [17,18]. GPR120 activation by unsaturated FAs particularly of the omega-3 variety prevents signaling through the IKK-β/NF-κB pathway by physically interacting with β2-arrestin and sequestering the major activator of the pathway, transforming growth factor-β-activated kinase 1 (TAK1) binding protein (TAB1) [19]. Without sufficient TAB1 available to activate its partner protein TAK1 the downstream kinase IKK-β and key transcription factor NFκB remain dormant and the transcriptional inflammatory response is not induced [19]. Despite the fact that GPR120 is well documented to signal through the Gαq/ll subunit activating the protein kinase C (PKC)- mitogen activated protein kinase (MAPK)- extracellular signal regulated kinase (ERK) (PKC-MAPK-ERK) and the phosphoinositide 3-kinase (PI3K)-AKT (protein kinase b) cascades, the relevance of either cascade in mediating the anti-inflammatory properties of GPR120 remains unexplored [18,19].

The presence of functional GPR120 and adequate omega-3 FAs for its activation is sufficient to lower systemic inflammatory state and improve overall energy utilization in mice [19]. Genetic disruption of GPR120 eliminates the ability of dietary omega-3 FAs to improve energy homeostasis in obese mice, attesting to the potency of this receptor [19]. Importantly, GPR120 activity is also physiologically relevant in humans given the recent discovery of functionally disrupted GPR120 mutations in obese Europeans [20]. The anti-inflammatory actions of GPR120 in response to long-chain FAs, in particular omega-3 FAs, have been elegantly demonstrated in adipocytes and macrophages, but little is known regarding these actions in other tissues throughout the body. Recently, GPR120 expression and the GPR120-β2-arrestin-TAB1 interaction were identified in the hypothalamus, but its role in the CNS remains poorly understood [21]. Given the anti-inflammatory actions of GPR120 in the periphery and the conservation the IKK-β/NF-κB pathway in the CNS, we hypothesize that GPR120 may modulate hypothalamic function by altering the inflammatory status of this tissue. We test this hypothesis at the cellular level using a hypothalamic neuronal model, rHypoE-7, isolated from the rat.

## Methods

### Cell culture

The hypothalamic cell line from the embryonic rat, rHypoE-7, was isolated and immortalized as previously described [22,23]. Cell lines were maintained and grown to confluency in Dulbecco’s Modified Eagle Medium (DMEM) (Sigma, St. Louis MO, USA) supplemented with 5% fetal bovine serum (FBS), 1% penicillin, and 1% streptomycin (GIBCO, Big Cabin, OK, USA) and seeded into 60 mm culture plates 24 hrs prior to experimental treatments.

### Production of cDNA and quantitative real-time reverse transcriptase-PCR

Total cellular RNA was isolated as previously described using the guanidium thiocyanate phenol chloroform extraction method [24]. Contaminating DNA was removed from all RNA samples by Turbo DNAase (Ambion, Austin, TX, USA) treatment (1 hr, 37°C). For quantitative real-time reverse transcriptase-PCR (qRT-PCR) experiments, cDNA was produced using the Applied Biosystems High Capacity cDNA Reverse Transcriptase kit and 50 ng of cDNA per sample was analyzed using an Applied Biosystems Prism 7000 Real-Time PCR machine together with gene-specific primers [See Additional file 1: Table S1] and SYBR green master mix.

### Semi-quantitative reverse transcriptase-PCR

DNAse treated RNA (200 μg) was subjected to one-step reverse transcriptase-PCR (RT-PCR) analysis (Qiagen, Mississauga, ON, CAN) according to manufacturer’s instructions and using primers listed in Additional file 1: Table S1. Product size and nucleotide sequencing confirmed the identities of all PCR products.

### Fatty acid, inflammatory, and anti-inflammatory treatments

For induction of the inflammatory response, rHypoE-7 cells were treated with tumor necrosis factor α (TNFα) (Sigma; 1 to 100 ng/mL) for 10 min for the assessment of protein phosphorylation levels or 2 to 6 hr for measurement of the mRNA response. For the anti-inflammatory experiments, cells were serum-starved for 1 hr and exposed to 100 μM docosahexaenoic acid (DHA) or GW9508 (4-(3-phenoxybenzylamino)-phenylpropionic acid) (Sigma) in DMSO for 1 hr prior to TNFα treatment. Unless otherwise indicated, all inhibitor studies were done by pretreating cells for 1 hr prior to DHA exposure. These inhibitors which include N-(6-Chloro-9H-pyrido[3,4-b]indol-8-yl)-3-pyridinecarbox-amide dihydrochloride (PS1145; 20 μM), staurosporine aglycone (1 μM), and Wortmannin (1 μM) were purchased from Sigma. For experiments involving DHA-bovine serum albumin (BSA) complexes, BSA and DHA were co-incubated at identical concentrations for 1 hr prior to their use.

### Co-immunoprecipitation

Cells were treated with DMSO or DHA (100 μM) for 30 min prior to being lysed in radioimmunoassay (RIPA) buffer supplemented with 0.2% (v/v) SDS, 0.1% (v/v) Triton X-100 and 1 mM PMSF (Sigma). The soluble fraction was incubated with the anti-TAB1 antibody (1 μg; Abcam, Cambridge, MA, USA) overnight at 4°C and for an additional hour with equilibrated protein A/G sepharose beads (Santa Cruz). The beads were washed three times with RIPA buffer supplemented with SDS and Triton X-100 as above, and protein complexes were eluted into sample buffer (1 M Tris-Cl, 8% (w/v) SDS, 40% (v/v) glycerol, 50 mM EDTA, 4% (v/v) β-mercaptoethanol, and 1 mM bromophenol blue) by boiling.

### Protein isolation, SDS-PAGE, and western blotting

After two washes with phosphate buffered saline (PBS), cells were scrapped on ice into lysis buffer (Cell Signaling Technology Inc., Danvers, MA, USA) supplemented with 1 mM PMSF and the soluble fraction was isolated after centrifugation (14000 rpm, 10 min, 4°C). Protein was quantified using a BCA Protein Assay Kit (Thermo Scientific, Rockford, IL, USA) according to manufacturer’s protocol, and lysates were resolved on 8% poly-acrylamide gels and transferred onto Immobilon-P PVDF membrane (Bio-Rad, Hercules, CA, USA). Membranes were blocked in 5% milk in Tris buffered saline with 0.1% Tween-20 (TBST) for 1 hr, followed by an overnight incubation at 4°C in primary antibody (1:1000). Membranes were washed with TBST before and after exposure to goat-anti-rabbit HRP secondary antibody (1 hr; Cell Signaling) and protein were visualized using Kodak 1D Image Analysis Software 3.6 and a Kodak Image Station 2000R (Eastman Kodak Company, Rochester, NY, USA). Primary antibodies used for western blotting include phospho-p44/42 MAPK (ERK1/2), p44/42 MAPK (ERK1/2), phospho-AKT, AKT, phospho-JNK, phospho-IKK, phospho-NF-κB, phospho-elF-2α, and phospho-TAK1 were obtained from Cell Signaling, anti-GPR120 was obtained from Abcam and anti-β-actin and anti-TNFα were obtained from Sigma.

### MTT assay

Cell viability was assessed using the MTT 3-(4,5-dimethylthiazol-2-yl)-2,5-diphenyl-tetrazolium bromide method after TNFα treatment using a Vybrant MTT Cell Proliferation Assay Kit (Invitrogen, Eugene, OR, USA) according to the manufacturer’s protocol. Briefly, cells were plated into a 96 well plate, treated with 10 μM palmitate for 2 hr, and incubated for 4 hr at 37°C with 1 mM MTT reagent. Formazan produced was solublized in DMSO and quantified by measuring the absorbance at 570 nm. All values are normalized to solvent controls.

### Knockdown of GPR120 by siRNA

The rHypoE-7 cells were plated into 6 well plates and GPR120 mRNA and protein were knocked down using Dharmafect transfection reagent (Thermo Scientific) and a TriFECTa™ Kit (Integrated DNA Technology, Coralville, Iowa, USA) containing a non-targeting DsiRNA 5′-GGU AAA CCA UGG UGU GC-3′ and antisense 5′-CUU UAC AUG CAC ACC AUG-3 and three DsiRNA oligonucleotide sets specifically targeting rat GPR120: GPR120a sense 5′-GGA CCA GCA AAU UAA GGA ACG AUC G-3′ and antisense 5′-CGA UCG UUC CUU AAU UUG CUG GUC CUG-3′, GPR120b sense 5′-CCC AAC CGC AUA GGA GAA AUC UCA T-3′ and antisense 5′-AUG AGA UUU CUC CUA UGC GGU UGG GCC-3′, GPR120c sense 5′-GGU AAA CCA UGG UGU GCA UGU AAA G-3′and antisense 5′-CUU UAC AUG CAC ACC AUG GUU UAC CUG-3′. Dharmafect (1 nM) and siRNA (20 ng) complexes were added to the rHypo-7 cells 24 hr and GPR120 mRNA and protein was quantified 24 hr after transfection. The GPR120c set of DsiRNAs was the most effective in reducing the mRNA levels of GPR120 within the rHypoE-7 cell line and thus this set was used in all experiments where endogenous GPR120 levels were reduced.

### Densitometry and statistics

Pixel intensity was quantified using the ImageJ program (U.S. National Institutes of Health, Bethesda, Maryland, USA, http://imagej.nih.gov/ij/, 1997-2014) and all statistics were calculated using Graphpad prism 5.0 (San Diego, CA, USA). Groups were compared by a two-tailed t-test or by a one-way or two-way ANOVA with the Bonferroni test for post hoc comparisons where appropriate. Data are presented as the mean ± SEM and the significance is denoted by *P <0.05, **P <0.01, ***P <0.001.

## Results

### rHypoE-7 cell line expresses key components of the inflammatory cascade, IKK-β/NF-κB

**Figure 1:**
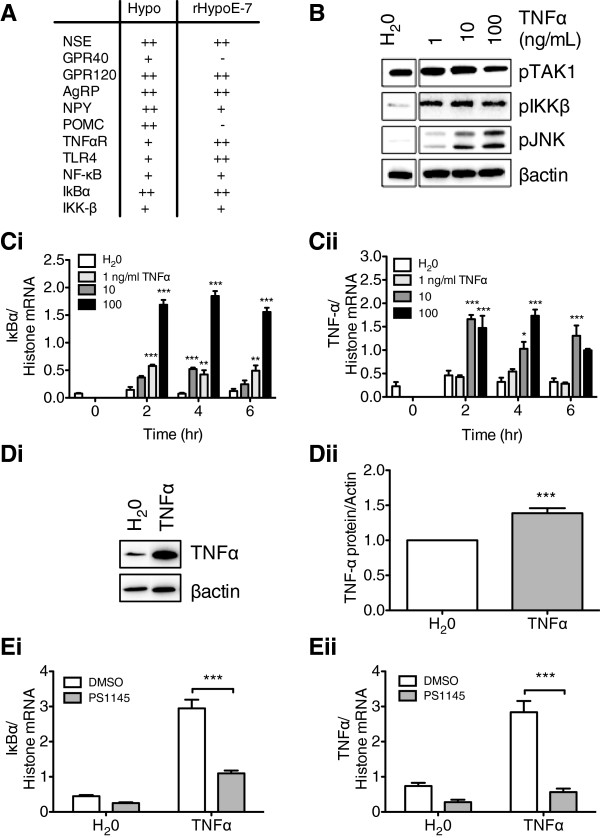
**TNFα activates the inflammatory IKK-β/NF-κB cascade in rHypoE-7 cells. A**. Summary of gene transcripts detected in the hypothalamus and rHypoE-7 cell line by semi-quantitative reverse transcriptase-PCR (RT-PCR) (transcript absent (-), present (+), or abundant (++); neuron specific enolase (NSE), G-protein coupled receptor (GPR) 40 (GPR40), GPR120, Agouti-related neuropeptide (AgRP), pro-opiomelancortin (POMC), tumor necrosis factor alpha receptor (TNFαR), toll-like receptor *4 (TLR4), nuclear factor kappa B (NF*κ*B), NF*κ*B inhibitor alpha (I*κ*B*α*), IkappaB kinase beta (IKK-*β*)*. **B**. Western blots showing activation and thus phosphorylation of TAK1 (pTAK1), IKK-β (pIKK-β), and JNK (pJNK) upon TNFα treatment (1 to 100 ng/mL, 10 min) or H_2_0 control relative to β-actin. **C**. Bar graph showing the mRNA levels of IκBα **(i)** and TNFα **(ii)** after vehicle (H_2_0) or TNFα treatment (1 to 100 ng/mL, 2 to 6 hr, n = 3 to 7). **D (i)**. Western blot showing TNFα protein production after vehicle (H_2_0) or TNFα exposure (10 ng/mL, 2 hr) in serum-free media followed by a 6-hr recovery incubation in media with FBS. **(ii)**. Production of TNFα protein was significantly enhanced relative to vehicle (H_2_0) control (1.39 ± 0.07 TNFα/βactin protein, n = 4). **E**. Pretreatment of rHypoE-7 cells with the IKK-β inhibitor, PS1145 (20 μM, 1 hr) prior to the addition of TNFα (10 ng/mL, 2 hr) significantly reduced the transcriptional inflammatory response as shown by a reduced mRNA level of IκBα **(i)** (DMSO; white bar: 2.95 ± 0.25 and PS1145; gray bar: 1.10 ± 0.08 IκBα/histone mRNA, n = 7) and TNFα **(ii)** (DMSO; white bar: 2.84 ± 0.32 and PS1145; gray bar: 0.56 ± 0.10 TNFα/histone mRNA, n = 7). Data are shown as mean ± SEM; *P <0.05; **P <0.01; ***P <0.01).

Generation of the rHypoE-7 cell line from the embryonic rat hypothalamus was completed as previously described and semi-quantitative RT-PCR was used to characterize the gene expression of numerous markers, neuropeptides, receptors, and signal transduction components [22,23]. Specifically, the rHypoE-7 cell line was screened for mRNA transcripts encoding the free FA sensing G protein coupled receptors (GPRs; GPR-40 and GPR-120), the neuronal marker neuron-specific enolase (NSE), hypothalamic neuropeptides (AgRP, NPY, and POMC), and inflammatory receptors and cytokines (Figure 1A). Consistent to their origin rHypoE-7 cells expressed NSE and hypothalamic neuropeptides including AgRP and NPY (Figure 1A). Furthermore mRNA transcripts encoding various cytokine receptors including the TNFα receptor 2 (TNFαR), interleukin (IL)-1β receptor (IL-1Rec), and IL-6 receptor (IL-6Rec), as well as IKK-β/NF-κB cascade components, were also identified, indicating that the rHypoE-7 neuronal model has the sufficient machinery to illicit an innate immune response against the pro-inflammatory cytokines. For this study inflammation was induced in rHypoE-7 cell model by exposure to the pro-inflammatory cytokine, TNFα.

### TNFα activates the IKK-β/NF-κB in rHypoE-7 cells

In order to determine if the rHypoE-7 cell line is capable of eliciting an innate immune response to inflammatory cytokines, we assessed the activation of IKK-β/NF-κB cascade upon TNFα exposure using western blotting and qRT-PCR. Essentially, treatment of rHypoE-7 cells with TNFα (1 to 100 ng/mL) dissolved in H_2_0 led to a rapid (10 min) increase in phosphorylation levels of IKK-β, and JNK (Figure 1B) relative to the vehicle control (H_2_0). A modest increase in the phosphorylation levels of the transforming growth factor-β-activated kinase 1 (TAK1) was also observed up to 10 ng/mL TNFα. Furthermore, TNFα treatment led to an upregulation of mRNA levels of IκBα and TNFα at 2 to 6 hr (Figure 1Ci, ii). Treatment with 10 ng/mL TNFα (2 hr) was sufficient to dramatically enhance TNFα mRNA relative to control (1.66 ± 0.09 and 0.46 ± 0.10 TNFα/histone mRNA, respectively) and TNFα protein levels relative to vehicle (1.39 ± 0.07 TNFα/β-actin protein) within this cell line (Figure 1Di, ii). To ensure that the upregulation of TNFα mRNA and protein was the result of the activation of the specific IKK-β/NF-κB cascade, the rHypoE-7 cell line was treated with the IKK-β inhibitor, PS1145 prior to TNFα exposure [15]. As predicted 20 μM PS1145 was sufficient in reducing the transcriptional inflammatory response of IκBα (DMSO: 2.95 ± 0.25 and PS1145: 1.10 ± 0.08 IκBα/histone mRNA) and TNFα (DMSO: 2.84 ± 0.32 and PS1145: 0.56 ± 0.10 TNFα/histone mRNA) (Figure 1Ei, ii). Together, these results indicate that the canonical inflammatory pathway is functionally conserved in the rHypoE-7 cell model. Given that an effective transcriptional and translational inflammatory response was observed upon treatment with TNFα at 10 ng/mL for 2 hr, this incubation time and concentration was used in all subsequent studies.

### TNFα exposure did not activate endoplasmic reticulum stress or apoptosis

**Figure 2:**
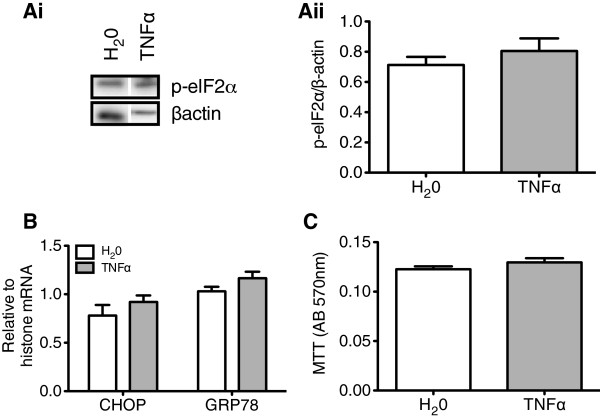
**TNFα activates the IKK-β/NF-κB cascade without significant induction of endoplasmic reticulum (ER) stress or apoptosis in rHypoE-7 cells. Ai**. The phosphorylation levels of the ER stress marker, elF-2α, were assessed by western blotting after TNFα treatment (10 ng/mL, 2 hr). Relative to vehicle (H_2_0) and normalized to β-actin, no significant increase in phospho-elF2α (p-elf2α) was observed **(ii)** (H_2_0: 0.71 ± 0.05 and TNFα: 0.81 ± 0.08 p-elF-2α/βactin, n = 4). **B**. ER stress levels were also assessed by measuring the mRNA levels of ER stress markers, CHOP and GRP-78, by qRT-PCR (n = 4-10). No significant change was detected relative to vehicle (H_2_0) treatment for either CHOP (H_2_0; white bar: 0.78 ± 0.11 and TNFα; gray bar: 0.92 ± 0.07 CHOP/histone mRNA, n = 4 to 10) or GRP78 (H_2_0: 1.03 ± 0.05 and TNFα: 1.17 ± 0.07 GRP78/histone). **C**. Bar graph representing the total MTT absorbance (AB_570nm_) upon H_2_0 or TNFα treatment (10 ng/mL, 2 hr) where no significant change in absorbance could be detected (H_2_0; white bar: 0.12 ± 0.002 and TNFα; gray bar: 0.13 ± 0.004 AB_570nm_, n = 8 to 16). Data are shown as mean ± SEM.

As the IKK-β/NF-κB cascade has been associated with the induction of endoplasmic reticulum (ER) stress or apoptotic cascades both pathways were examined in order to fully characterize the inflammatory state of rHypoE-7 cells [25]. Relative to H_2_0 treatment alone, TNFα did not increase the phosphorylation levels of the ER transcription factor, elongation factor 2-α (p-elF-2α; 0.71 ± 0.05 and 0.81 ± 0.08 p-elF-2α/β-actin, respectively) or the mRNA levels of the ER chaperone, GRP-78 (glucose responsive protein-78; 1.03 ± 0.05 and 1.17 ± 0.07 GRP78/histone mRNA, respectively) or transcription factor CHOP (C/EBP homologous protein; 0.78 ± 0.11 and 0.92 ± 0.07 CHOP/histone mRNA, respectively) (Figure 2A, B). It should be noted that a longer exposure to a higher TNFα concentration (6 hr, 100 ng/mL) elevated CHOP mRNA levels suggesting ER stress may come into play later in the inflammatory process (data not shown). Activation of apoptotic pathways was also ruled out by the MTT assay, which revealed an identical cell number/and or metabolic activity across treatments (H_2_0: 0.12 ± 0.002 and TNFα: 0.13 ± 0.004 AB_570nm_) (Figure 2C). Together with the findings in Figure 1, an acute exposure to modest TNFα concentrations is sufficient to produce a robust transcriptional and translational inflammatory response, without accompanying ER stress or apoptosis. This finding will enable us to specifically examine activation of the IKK-β/NF-κB pathway in the rHypoE-7 neuronal model.

### Docosahexaenoic acid (DHA) pretreatment prevents TNFα-dependent inflammation in rHypoE-7 cells

**Figure 3:**
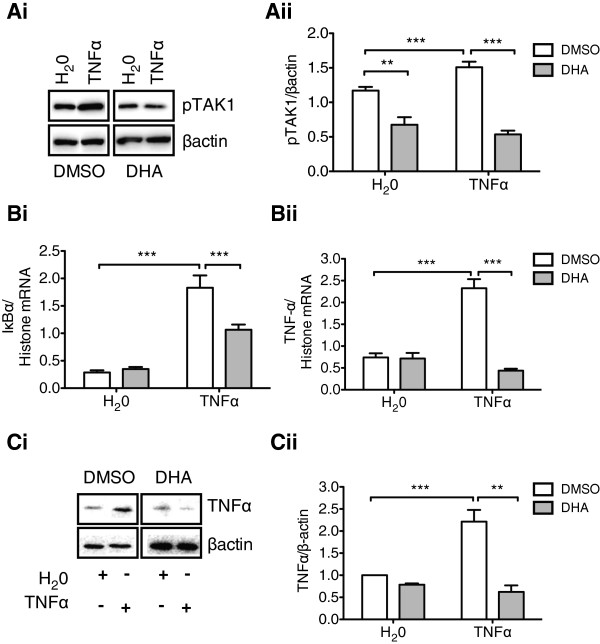
**Docosahexaenoic acid (DHA) pretreatment inhibits the pro-inflammatory response to TNFα in rHypoE-7 cells. A (i)**. Western blots (α-phospho-TAK1 (pTAK1) and α-β-actin) of rHypoE-7 cells pretreated with 100 μM DHA or vehicle (DMSO) for 1 hr prior to the addition of 10 ng/mL TNFα for 10 min. **(ii)**. DHA pretreatment (gray bars) prior to TNFα exposure significantly reduced pTAK1 levels relative to DMSO alone (white bars) (0.54 ± 0.06 and 1.51 ± 0.08 pTAK1/βactin, respectively). **B**. Relative to DMSO (white bars), DHA (gray bars) pretreatment (100 μM, 1 hr) significantly reduced the inflammatory transcriptional response to TNFα (10 ng/mL, 2 hr) as shown from mRNA levels of IκBα **(i)** (DMSO: 1.83 ± 0.22 and DHA: 1.07 ± 0.09 IκBα/histone mRNA, n = 4) and TNFα **(ii)** (DMSO: 2.33 ± 0.21 and DHA: 0.44 ± 0.04 TNFα/histone mRNA, n = 4). **C (i)**. Western blots (α-TNFα, α-βactin) demonstrating TNFα protein levels in rHypoE-7 cells treated as described in Section B, with an additional recovery incubation (6 hr) in media containing FBS. As with mRNA levels, DHA pretreatment (gray bars) significantly reduced TNFα protein production relative to the DMSO vehicle control (white bars) (DMSO: 2.21 ± 0.26 and DHA: 0.62 ± 0.30 TNFα/βactin; n = 3 to 4) **(ii)**. Data are shown as mean ± SEM; **P <0.01; ***P <0.01).

To determine if long-chain omega-3 FAs exhibit an anti-inflammatory property in rHypoE-7 cells, we pretreated the cells with 100 μM FAs or DMSO vehicle 1 hr prior to exposure to TNFα (10 ng/mL, 10 min) and monitored activation of the IKK-β/NF-κB cascade by Western blotting. As DHA is the most abundant omega-3 FA in the brain, we focused on using this FA in our studies [26]. DHA pretreatment reduced phospho-TAK1 and phospho-NF-κB levels relative to DMSO control, suggesting this FA can inhibit signaling through the IKK-β/NF-κB cascade (Figure 3A). DHA exposure lowered the level of active TAK1 relative to DMSO even without an inflammatory challenge, suggesting that the rHypoE-7 cell model may exhibit a lower basal level of signaling through the IKK-β/NF-κB pathway.

Furthermore, DHA pretreatment significantly abrogated the TNFα-dependent increase in mRNA levels of both proinflammatory markers: IκBα (DMSO: 1.83 ± 0.22 and DHA: 1.07 ± 0.09 IκBα/histone mRNA) and TNFα (DMSO: 2.33 ± 0.21 and DHA: 0.44 ± 0.04 TNFα/histone mRNA) (Figure 3Bi, ii). As expected from the mRNA results, DHA pretreatment also significantly reduced TNFα protein production and thus the translational inflammatory response (Figure 3C). These findings ensure that DHA exhibits anti-inflammatory properties in the hypothalamic rHypoE-7 cell model.

### The anti-inflammatory effects of (DHA) are AKT- and ERK-independent

**Figure 4:**
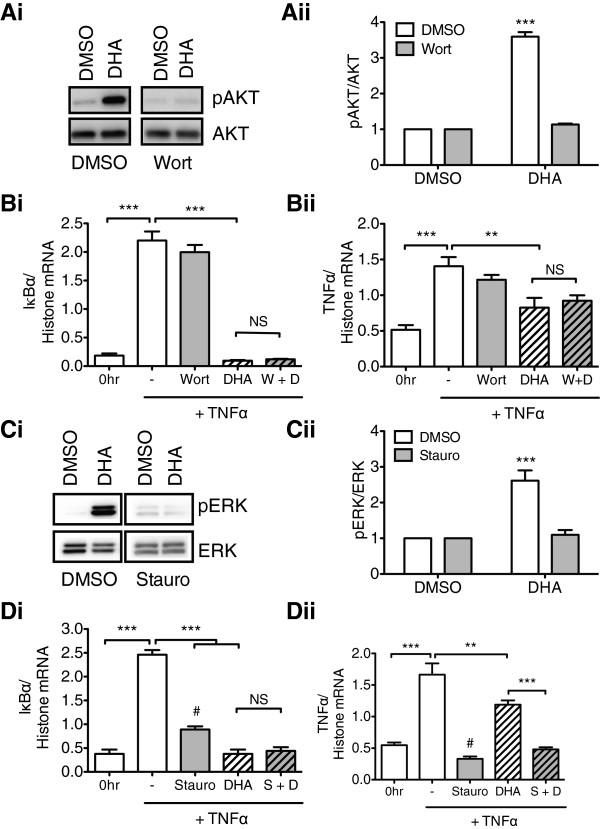
**Anti-inflammatory effects of docosahexaenoic acid (DHA) are AKT-independent and ERK-independent. Ai**. Treatment of rHypoE-7 cells with DHA (100 μM, 1 hr) caused an increase in phospho-AKT (pAKT)/AKT levels relative to DMSO alone and was abolished upon pre-treatment with 1 μM Wortmannin (Wort) **(ii)** (DHA; white bar: 3.60 ± 0.13 and DHA + Wort; gray bar: 1.13 ± 0.03 pAKT/AKT). **B**. Pretreatment with DMSO (-), Wort, DHA, or Wort with DHA (W + D) for 1 hr prior to exposure to TNFα (10 ng/mL, 2 hr) and mRNA levels of IκBα **(i)** (DHA; white striped bar: 0.09 ± 0.01 and Wort + DHA (W + D); gray stripped bar: 0.12 ± 0.01 IκBα/histone mRNA) and TNFα **(ii)** (DHA: 0.83 ± 0.14 and W + D: 0.92 ± 0.08 TNFα/histone mRNA) were assessed. Basal levels of IκBα and TNFα mRNA without TNFα treatment are shown (0 hr). **Ci**. Treatment with 100 μM DHA enhanced phospho-ERK (pERK)/ERK levels relative which was abolished upon 1 μM Staurosporine aglycone (Stauro) pretreatment **(ii)** (DHA: 2.61 ± 0.29 and DHA + Stauro: 1.36 ± 0.28 pERK/total ERK). **D**. Stauro pretreatment abolished or reduced the effect of IκBα **(i)** (#; DMSO (-); white bar: 2.46 ± 0.10 and Stauro; gray bar: 0.89 ± 0.07) or TNFα **(ii)** (#; DMSO (-); white bar: 1.66 ± 0.18 and Stauro; gray bar: 0.33 ± 0.04) respectively.. Stauro pretreatment did not impair the ability of DHA to reduce IκBα mRNA in response to TNFα exposure (DHA; white stripped bar: 0.38 ± 0.17 and Stauro + DHA (S + D); gray stripped bar: 0.44 ± 0.08 IκBα/histone mRNA) but further reduced TNFα mRNA levels (DHA; white stripped bar: 1.19 ± 0.07 and S + D; gray stripped bar: 0.48 ± 0.03 TNFα/histone mRNA). Data are shown as mean ± SEM; **, p < 0.01; *** p < 0.01; non-significant (NS)).

To determine if the anti-inflammatory actions of DHA is mediated through the phosphoinositide 3-kinase (PI3K)/AKT (protein kinase b) or mitogen activated protein kinase (MAPK)/extracellular signal regulated kinase (ERK) pathways, rHypoE-7 cells were co-treated with DHA and their respective inhibitors prior to TNFα exposure. Pretreatment of rHypoE-7 cells with the PI3K inhibitor, Wortmannin (Wort) significantly reduced AKT phosphorylation (pAKT) levels in response to DHA (DHA: 3.60 ± 0.13 and DHA/Wort: 1.13 ± 0.03 pAKT/AKT) but failed to prevent the anti-inflammatory effects of DHA as shown from the analysis of IκBα mRNA (DHA: 0.09 ± 0.01 and Wort/DHA (W + D): 0.12 ± 0.01 IκBα/histone mRNA) and TNFα mRNA (DHA: 0.83 ± 0.14 and W + D: 0.92 ± 0.08 TNFα/histone mRNA) (Figure 4A, B). Treatment of the rHypoE-7 cells with the PKC inhibitor, Staurosporine algyone (Stauro) reduced ERK phosphorylation (DHA: 2.61 ± 0.29 and DHA/Stauro: 1.36 ± 0.28 pERK/ERK) indicating pERK formation upon DHA exposure is PKC-dependent (Figure 4C). Pretreatment with Stauro abolished or reduced the enhancement of IκBα or TNFα respectively (Figure 4Di, ii), indicating that PKC itself participates in the induction of the transcriptional inflammatory response to TNFα in the rHypoE-7 cell line (a comparison denoted by # in Figure 4D). Relative to DHA pretreatment alone, DHA and Stauro (D + S) co-pretreatment prior to TNFα exposure did not cause a further reduction in IκBα mRNA levels (DHA: 0.38 ± 0.17 and SD + S: 0.44 ± 0.08 IκBα/histone mRNA) but led to a further decrease in TNFα mRNA levels (DHA: 1.19 ± 0.07 and D + S: 0.48 ± 0.03 TNFα/histone mRNA (Figure 4D). These results indicate that the ability of DHA to activate PKC activity plays a role in its anti-inflammatory actions. These results suggest that although DHA activates PI3K and PKC, neither signaling cascade plays significant roles in mediating the anti-inflammatory actions of this omega-3 FA.

### GPR120 mediates the anti-inflammatory effect of DHA in rHypoE-7 cells

**Figure 5:**
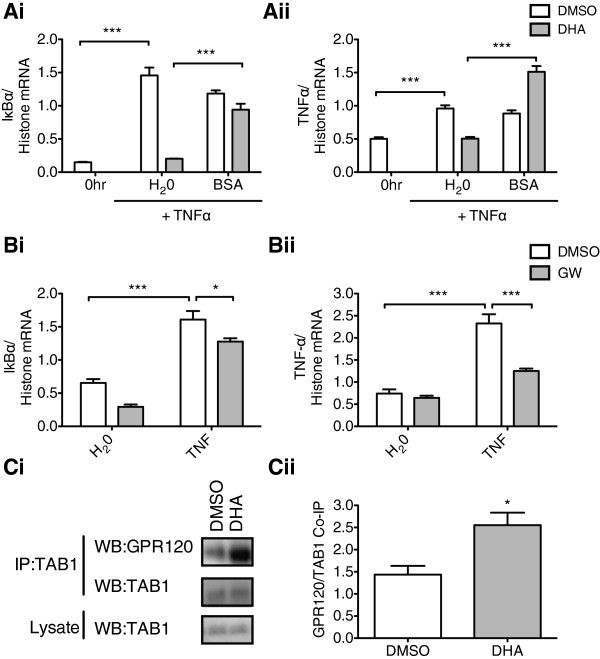
**The anti-inflammatory effect of docosahexaenoic acid (DHA) is abolished by the presence of BSA and replicated by the GPR120 agonist, GW9508. A**. The presence of BSA (100 μM in H_2_0) eliminated the anti-inflammatory effect of DHA pretreatment (100 μM in DMSO, 1 hr) on the transcriptional inflammatory response to TNFα treatment (10 ng/mL, 2 hr) as shown from the mRNA levels of IκBα **(i)** (DHA + H_2_0: 0.20 ± 0.003 and DHA + BSA: 0.94 ± 0.09 IκBα/histone mRNA) and TNFα **(ii)** (DHA + H_2_0; 0.51 ± 0.02 and DHA + BSA: 1.51 ± 0.09 TNFα/histone). **B**. The rHypoE-7 cells were pretreated with 100 μM GW9508 (GW) for 1 hr prior to the addition of 10 ng/mL TNFα and mRNA levels of IκBα **(i)** (DMSO; white bar: 1.61 ± 0.13 and GW; *gray bar*: 1.28 ± 0.05 IκBα/histone, n = 5 to 8) and TNFα **(ii)** (DMSO; white bar: 2.33 ± 0.21 and GW; gray bar: 1.31 ± 0.07 TNFα/histone, n = 5 to 8) were quantified by quantitative reverse transcriptase-PCR (qRT-PCR). GW significantly reduced the mRNA levels of both pro-inflammatory indicators relative to the DMSO vehicle. **C**. GPR120 co-immunoprecipitation (IP) with TAB1 shown by Western blots (WB) **(i)**. DHA pretreatment significantly increased GPR120-TAB1 association **(ii)** (DMSO; white bar: 1.44 ± 0.40 and DHA; *gray bar*: 2.55 ± 0.49 GPR120/TAB1; n = 3 to 4). Data are shown as mean ± SEM; *P <0.05; ***P <0.01).

The transcriptional anti-inflammatory actions of DHA in rHypoE-7 cells was abolished by the presence of BSA as shown upon quantifying mRNA levels of IκBα (DHA + H_2_0: 0.20 ± 0.003 and DHA + BSA: 0.94 ± 0.09 IκBα/histone mRNA) and TNFα, (DHA + H_2_0: 0.51 ± 0.02 and DHA + BSA: 1.51 ± 0.09 TNFα/histone) after TNFα exposure (Figure 5A). As the addition of BSA and the formation of DHA-BSA complexes abolished the ability of DHA to inhibit the transcriptional inflammatory response, it is clear that DHA must exist in its free form to mediate its acute anti-inflammatory actions and may interact with free FA receptors at the cell surface.

The free FA receptor, GPR120, was recently identified to mediate the anti-inflammatory actions of omega-3 FAs in macrophages, and to determine if GPR120 may be involved in the anti-inflammatory actions in the hypothalamic rHypoE-7 cell model, we employed the GPR120 synthetic agonist GW9508 (GW) [19,27]. It must be noted here that GW can also activate GPR40, but as GPR40 is not expressed in the rHypoE-7 cell model, this agonist is considered a GPR120-specific in our studies (Figure 1A). Pretreatment of rHypoE-7 cells with GW (100 μM, 1 hr) prior to TNFα exposure significantly reduced mRNA levels of inflammatory indicators, IκBα (DMSO: 1.61 ± 0.22 and GW: 1.28 ± 0.05 IκBα/histone mRNA) and TNFα (DMSO: 2.33 ± 0.21 and GW: 1.31 ± 0.07 TNFα/histone mRNA) (Figure 5B). This finding indicates that GPR120 activation exhibits anti-inflammatory properties in the rHypoE-7 cell line.

In addition to enhancing the levels of phospho-ERK and phospho-AKT, activated GPR120 can also associate with TAB1. This interaction prevents the subsequent activation of its partner protein TAK1 and halts signaling through the pro-inflammatory IKK-β/NFκB cascade [19]. To determine if this interaction is conserved in the hypothalamic cell model we treated rHypoE-7 cells with DMSO or DHA for 30 min and monitored the GPR120-TAB1 association by co-immunoprecipitation. DHA treatment increased the GPR120-TAB1 association relative to DMSO control indicating that a similar mechanism identified in the periphery is likely conserved in the hypothalamic cell model (DMSO: 1.44 ± 0.40 and DHA: 2.55 ± 0.28 GPR120/TAB1) (Figure 5C).

**Figure 6:**
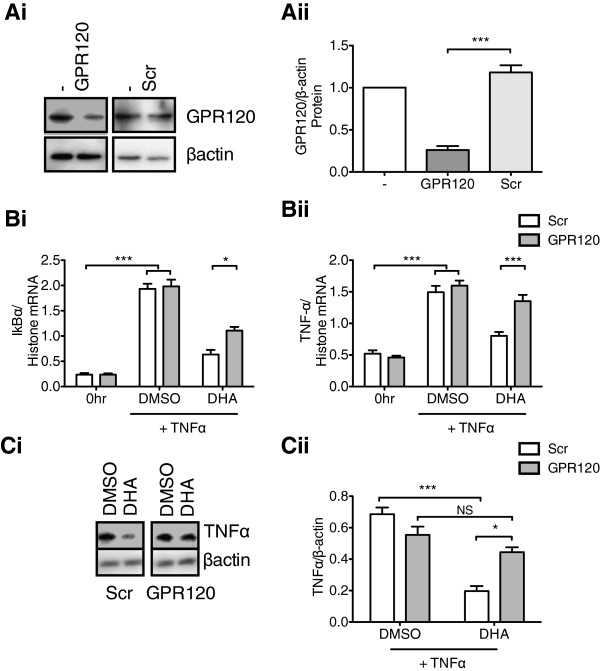
**Knockdown of GPR120 expression reduces the anti-inflammatory effect of docosahexaenoic acid (DHA). A (i)**. Western blot of rHypoE-7 cells treated with GPR120-specific or scrambled (Scr) siRNA for 24 hr showing that exposure to GPR120-specific siRNA reduced endogenous GPR120 protein levels by approximately 75% **(ii)**. Scr; light gray bar: 1.18 ± 0.08 and GPR120; dark gray bar: 0.26 ± 0.05 GPR120/β-actin, n = 4). **B**. GPR120 reduction significantly impaired the ability of DHA (100 μM, 1 hr pretreatment) to reduce IκBα **(i)** (Scr: 0.63 ± 0.09 and GPR120: 1.11 ± 0.07 IκBα/histone) and TNFα **(ii)** (Scr: 0.82 ± 0.06 and GPR120: 1.35 ± 0.10 TNFα/histone) mRNA levels upon TNFα treatment (10 ng/mL, 2 hr). **C**. Reduction of GPR120 levels by siRNA significantly impaired the ability of DHA to reduce TNFα protein production relative to Scr controls (Scr + DHA = 0.20 ± 0.03 and GPR120 + DHA = 0.44 ± 0.03 TNFα/β-actin, n = 3). Data are shown as mean ± SEM; *P <0.05; ***P <0.01; not significant (NS).

To reduce endogenous level of GPR120 in the rHypoE-7 neuronal model we used the small interfering RNA (siRNA) method and GPR120-specific oligonucleotides. Importantly, siRNA resulted in approximately 40% reduction in GPR120 mRNA (data not shown) and 75% reduction in protein levels as evident from qRT-PCR and Western blotting, respectively (Figure 6A). Reduction of endogenous GPR120 levels enabled us to directly test the role of this GPR in mediating the anti-inflammatory actions of DHA. Reduction of endogenous levels of GPR120 protein significantly impaired the anti-inflammatory actions of DHA as seen by an increase in inflammatory transcripts relative to control for IκBα (Scr: 0.63 ± 0.09 and GPR120: 1.11 ± 0.07 IκBα/histone mRNA) and TNFα (Scr: 0.82 ± 0.06 and GPR120: 1.35 ± 1.67 TNFα/histone mRNA) (Figure 6B). Despite the observation that DHA could lower TNFα protein production by roughly 70% in the Scr controls (DMSO = 0.63 ± 0.04 and DHA = 0.20 ± 0.03 TNFα/βactin), no significant difference could be observed upon a reduction in endogenous GPR120 levels (DMSO = 0.55 ± 0.05 and DHA = 0.44 ± 0.03 TNFα/βactin) (Figure 6C). Taken together, GPR120 is functionally active in the rHypoE-7 cell model, wherein its activation by DHA inhibits the transcriptional and translational inflammatory response against the pro-inflammatory cytokine TNFα.

## Discussion

Hypothalalmic inflammation disrupts energy homeostasis by leading to pathogenic changes in insulin signaling, feeding and body weight and thus is a major target for the prevention and treatment of various metabolic diseases including DIO and T2DM. Here we identify the omega-3 FA receptor GPR120 as an anti-inflammatory mediator in the hypothalamic neuronal model, rHypoE-7, isolated from the rat. Essentially, rHypoE-7 cells expressed sufficient machinery to undergo a transcriptional and translational inflammatory response to the pro-inflammatory cytokine, TNFα without a significant induction of ER stress or apoptotic pathways upon acute exposures, enabling the specific examination of the activity of the IKK-β/NF-κB cascade. Activation of GPR120 by DHA was sufficient in reducing the inflammatory response to TNFα at the transcriptional and translational levels. Disruption of endogenous GPR120 significantly aborted the anti-inflammatory effects of DHA identifying GPR120 as the prime mediator of omega-3 FA actions on the inflammatory status in this cell model. Taken together, the rHypoE-7 cell line is a sufficient model and valuable tool in the study of hypothalamic inflammation and the anti-inflammatory actions of GPR120 in response to omega-3 FAs at the neuronal and molecular levels.

Despite the evidence that GPR120 is a Gαq/ll GPR protein and can activate the PI3K/AKT and the PKC/MAPK/ERK cascades as shown here and by other groups, these signaling components are likely dispensable for its anti-inflammatory actions [18,19]. Instead GPR120 intercepts the inflammatory IKK-β/NF-κB pathway by scaffolding through β2-arrestin to key inflammatory modulators such as TAB1 to prevent the downstream activation of pro-inflammatory kinases and transcription factors [19]. To date, the GPR120-TAB1 interaction has been demonstrated in a wide variety of cell types including macrophages, monocytes, fibroblasts, adipocytes, and now hypothalamic neurons and neuronal models, and likely represents a highly conserved mechanism of anti-inflammatory action employed by omega-3 FAs [18,19,21,28,29]. In addition to the GPR120-TAB1 interaction, GPR120 has also been shown to scaffold to NLRP3 (nucleotide-binding domain and leucine-rich repeat containing protein) through β2-arrestin to prevent the formation of the NLRP3 inflammasome in an omega-3 FA-dependent manner [30]. The activation of the NLRP3 inflammasome is generally pathogen-based and likely represents a GPR120-dependent mechanism more critical in peripheral tissues than the hypothalamus, which is generally protected from such infections by the blood brain barrier. However, the recent discovery that β2-arrestin has the capacity to scaffold hundreds of different proteins to the parental GPR, it is likely that other GPR120 interactions mediating the anti-inflammatory response of omega-3 FAs will emerge with further investigation [31].

GPR120 protein expression was recently localized within the arcuate nucleus of the hypothalamus particularly in NPY neurons also found to express AgRP [21]. Interestingly, our neuronal model, rHypoE-7, also expresses both neuropeptides suggesting that GPR120 expression and its omega-3 dependent actions may be concentrated within this neuron population. This observation is intriguing considering that the ablation of the IKK-β/NF-κB cascade in AgRP/NPY neurons is sufficient to prevent diet induced diseases in high fat diets and puts forth the possibility that GPR120 may serve to provide the brake on inflammation that would otherwise lead to pathogenic consequences [14]. Given the orexigenic nature of the AgRP/NPY neuronal population, whether or not GPR120 could modulate neuropeptide expression or secretion and thus directly impact appetite and feeding is an obvious question. To date, we have not detected any changes in neuropeptide expression (at least at the mRNA level) upon DHA treatment with our GPR120-expression cell line, rHypoE-7 (unpublished results, LW and DB). However, mice deficient in GPR120 are more susceptible to weight gain upon high fat feeding, which has been attributed to lower rates of basal metabolism, heightened insulin resistance, and higher expression of genes connected to inflammation [19,20]. Although food intake was measured to be identical for GPR120-knockout mice and their wildtype counterparts, additional analysis may be warranted given the recent identification of GPR120 variants in obese Europeans [20].

In addition to GPR120, long-chain omega-3 FAs can also promote anti-inflammatory actions by metabolism-dependent mechanisms by serving as substrates for the formation of anti-inflammatory metabolites known as resolvins and protectins or by activating peroxisome proliferator-activated receptors gamma (PPARγ) pathways by their oxidized products. As the pretreatment period of DHA prior to the TNFα addition was relatively short in these studies (1 hr), it is unlikely that the production of resolvins and protectins would be sufficient to contribute to the anti-inflammatory actions of this omega-3 FA in the rHypoE-7 cell model. Production of these metabolites peaks at 24 hours after an inflammatory stimulus and likely serves as a secondary means of resolving inflammation when other mechanisms fail to adequately do so [32]. Furthermore, the rHypoE-7 cell line exhibits undetectable levels of lipoxygenase 5 (LOX5), an enzyme involved in the formation of resolvins as shown by qRT-PCR (data not shown) [33]. Although the rHypoE-7 cell model does express PPARγ (data not shown) and thus has the ability to mediate PPARγ-dependent reduction in of inflammation by oxidative DHA products, this situation is unlikely to come into play during our short experimental protocol as DHA oxidative products peak at 10 hours after the inflammatory response [34]. Although a subset of unesterified DHA used in our treatments is likely incorporated into the phospholipid pool, this esterified population has been recently shown to lose its anti-inflammatory properties and thus likely does not significantly impact our study [35]. Future experiments using PPARγ or LOX inhibitors will reveal the impact of DHA metabolites on the acute anti-inflammatory response in rHypoE-7 cell model.

## Conclusions

Collectively, we use the hypothalamic neuronal model isolated from the rat hypothalamus, rHypoE-7 as a model of hypothalamic inflammation. The rHypoE-7 cell line exhibits an active canonical inflammatory cascade, IKK-β/NF-κB and can undergo an inflammatory response both at the transcriptional and translational level in response to the proinflammatory cytokine TNFα. Pretreatment with the omega-3 FA DHA inhibits the inflammatory response by enhancing the association between GPR120 and TAB1. Reduction of endogenous GPR120 protein levels was sufficient in abrogating the anti-inflammatory effects of DHA identifying GPR120 as the key mediator of the acute anti-inflammatory effects of DHA in this cell line. Future work will examine the impact of GPR120 in insulin sensitivity and orexigenic neuropeptide expression, such as NPY and AgRP, in the rHypoE-7 hypothalamic cell model.

## Abbreviations

AgRP: agouti-related neuropeptide; AKT: protein kinase B; BBB: blood-brain barrier; BSA: bovine serum albumin; CHOP: C/EBP homologous protein; DHA: docosahexaenoic acid; DIO: diet-induced obesity; ER: endoplasmic reticulum; ERK: extracellular signal regulated kinase; FA: fatty acid; GPR120: G-protein coupled receptor 120; GRP-78: glucose responsive protein-78; IKK-β/NF-κB: inhibitor of IkappaB kinase beta/nuclear factor kappa B; IL-1Rec: interleukin 1 receptor; IL-6Rec: interleukin 6 receptor; LOX5: lipoxygenase 5; NLRP3: nucleotide-binding domain and leucine-rich repeat containing protein 3; NPY: neuropeptide Y; p-elF-2α: elongation factor 2-α; NSE: neuron-specific enolase; PI3K: phosphoinositide 3-kinase; POMC: pro-opiomelancortin; PPARγ: peroxisome proliferator-activated receptors gamma; Stauro: staurosporine algyone; TAB1: TAK1 binding protein 1; TAK1: transforming growth factor-β-activated kinase 1; TNFα: tumor necrosis factor α; TNFαR: TNFα receptor 2; T2DM: type 2 diabetes mellitus; Wort: Wortmannin.

## Competing interests

The authors declare that they have no competing interests.

## Authors’ contributions

LW carried out all experimental techniques, statistical analysis, and drafted the manuscript. DDB conceived the study, and participated in its design and coordination, and helped to edit the manuscript. Both authors have read and approved the final manuscript.

## Supplementary Material

Additional file 1 Primers used for screening of markers. Click here for file
