# Supplementary material for: Activation of the omega-3 fatty acid receptor GPR120 mediates anti-inflammatory actions in immortalized hypothalamic neurons
Source: J Neuroinflammation. 2014 Mar 27;11:60. doi: 10.1186/1742-2094-11-60 (PMC3986641; doi:10.1186/1742-2094-11-60)
Supplement: Additional file 1 — Primers used for screening of markers. [file 1742-2094-11-60-S1.pdf]

Supplemental Table 1 – Primers used for screening of markers.

	Primer Sequence
**Histone 3a**	**Forward: 5' - CGC TTC CAG AGT GCA GCT ATT - 3'**
	**Reverse: 5' - ATC TTC AAA AAG GCC AAC CAG AT - 3'**
**NF-κB**	**Forward: 5' - AGT GAC AGC GAC AGT GAC AAC AGA - 3'**
	**Reverse: 5' - TCA TCA GGA AGA GGT TTG GCT GCT - 3'**
**TNFα**	**Forward: 5' - GCT GTA CCT TAT CTA CTC CC - 3'**
	**Reverse: 5' - CTC CTG GTA TGA AAT GGC - 3'**
**IKK-β**	**Forward: 5' - TTG GCA TCA CAT CGG ACA AAC TGC - 3'**
	**Reverse: 5' - TCT GCA GTG CCA TCA TCC GTT CTA - 3'**
**TLR4**	**Forward: 5' - GCT TGA ATC CCT GCA TAG - 3'**
	**Reverse 5' - GCT CAG ATC TAG GTT CTT GG - 3'**
**IkBα**	**Forward: 5' - TGC CTG GCC AGT GTA GTC TT - 3'**
	**Reverse: 5' - CAA AGT CAC CAA GTG CTC CAC GAT - 3'**
**TNFα Rec**	**Forward: 5' - TAG CCA AAC TCC ACA CAT CCC TGT -3'**
	**Reverse: 5' - ACA TCA GCA GAC CCA GAG TTG TCA - 3'**
**GRP-78**	**Forward: 5' - GAT GAA GTT CAC TGT GGT GGC GG - 3'**
	**Reverse: 5' - AGT CAA TGC CAA CCA CCA TGC CTA - 3'**
**CHOP**	**Forward: 5' - TAT GAG GAT CTG CAG GAG - 3'**
	**Reverse: 5' - CAG GGT CAA GAG TAG TGA AG - 3'**
**AgRP**	**Forward: 5' - CGG AGG TGC TAG ATC CAC AGA - 3'**
	**Reverse: 5' - AGG ACT CGT GCA GCC TTA CAC - 3'**
**NPY**	**Forward: 5' - TAG GTA ACA AGC GAA TGG GG - 3'**
	**Reverse: 5' - ACA TGG AAG GGT CTT CAA GC - 3'**
**POMC**	**Forward: 5' -TAG ATG TGT GGA GCT GGT GC - 3'**
	**Reverse: 5' - CAG TCA GGG GCT GTT CAT CT - 3'**
**NSE**	**Forward: 5' - CTG ATG CTG GAG TTG GAT G - 3'**
	**Reverse: 5' - CTT CGC TGT TCT CCA GGA TAT - 3'**
**GPR40**	**Forward: 5' - CGC TGG GCT TTC CAT TGA ACT TGT - 3'**
	**Reverse: 5' - ATG TTG ATG CCC AGG GAA CTG GTA - 3'**
**GPR120**	**Forward: 5'- TCT ACG TGA TGA CAA TGA GCG GCA - 3'**
	**Reverse: 5' - TCC GCG ATG CTT TCG TGA TCT GTA - 3'**
**IL-1Rec**	**Forward: 5'- ACA TCC ATG GGA GAT GCA GGC TAT- 3'**
	**Reverse: 5' - ATC ACA GGG ATG GGT TCT GTG GTT- 3'**
**IL-6Rec**	**Forward: 5'- TCA CTG TGC GTT GCA AAC AGT GTC- 3'**
	**Reverse: 5' - ATA CCA CAA GGT TGG CAG GTG GAT- 3'**
